# Native Liquid Chromatography and Mass Spectrometry to Structurally and Functionally Characterize Endo-Xylanase Proteoforms

**DOI:** 10.3390/ijms23031307

**Published:** 2022-01-24

**Authors:** Guusje van Schaick, Nadi el Hajjouti, Simone Nicolardi, Joost den Hartog, Romana Jansen, Rob van der Hoeven, Wim Bijleveld, Nicolas Abello, Manfred Wuhrer, Maurien M. A. Olsthoorn, Elena Domínguez-Vega

**Affiliations:** 1 Center for Proteomics and Metabolomics, Leiden University Medical Center, Albinusdreef 2, 2333 ZA Leiden, The Netherlands; nelhajjouti@outlook.com (N.e.H.); s.nicolardi@lumc.nl (S.N.); m.wuhrer@lumc.nl (M.W.); e.Dominguez_Vega@lumc.nl (E.D.-V.); 2 Center for Analytical Innovation, DSM, Alexander Fleminglaan 1, 2613 AX Delft, The Netherlands; Joost.Hartog-den@DSM.COM (J.d.H.); romana.jansen@DSM.COM (R.J.); Rob.Hoeven-van-der@DSM.COM (R.v.d.H.); Wim.Bijleveld@DSM.COM (W.B.); Nicolas.Abello@dsm.com (N.A.); Maurien.Olsthoorn@DSM.COM (M.M.A.O.)

**Keywords:** native protein analysis, endo-1,4-β-xylanase, boronate affinity chromatography, ion exchange chromatography, size exclusion chromatography, mass spectrometry, glycation

## Abstract

Xylanases are of great value in various industries, including paper, food, and biorefinery. Due to their biotechnological production, these enzymes can contain a variety of post-translational modifications, which may have a profound effect on protein function. Understanding the structure–function relationship can guide the development of products with optimal performance. We have developed a workflow for the structural and functional characterization of an endo-1,4-β-xylanase (ENDO-I) produced by *Aspergillus niger* with and without applying thermal stress. This workflow relies on orthogonal native separation techniques to resolve proteoforms. Mass spectrometry and activity assays of separated proteoforms permitted the establishment of structure–function relationships. The separation conditions were focus on balancing efficient separation and protein functionality. We employed size exclusion chromatography (SEC) to separate ENDO-I from other co-expressed proteins. Charge variants were investigated with ion exchange chromatography (IEX) and revealed the presence of low abundant glycated variants in the temperature-stressed material. To obtain better insights into the effect on glycation on function, we enriched for these species using boronate affinity chromatography (BAC). The activity measurements showed lower activity of glycated species compared to the non-modified enzyme. Altogether, this workflow allowed in-depth structural and functional characterization of ENDO-I proteoforms.

## 1. Introduction

Xylanases are industrially produced enzymes with a broad application in the paper and pulp, textile, food, and biorefinery industries [1]. For instance, xylanases are used to bleach pulp, generate biological fuels from lignocellulosic biomass, or improve dough properties during baking [2,3,4,5]. The biotechnological production of xylanases is often accomplished by microorganisms, such as bacteria or fungi [6,7,8]. In particular, the filamentous fungi *Aspergillus* and *Trichoderma* are known to produce high xylanase levels, making them suitable for commercial production [3]. Xylanases exist in many sizes varying from 6 to 80 kDa, with optimal performance between pH 3.0 and 6.5 and at temperatures of between 25 and 60 °C [4]. These enzymes often contain a plethora of post-translational modifications (PTMs), including glycosylation, disulfide bond formation, and proteolytic truncation [5]. Moreover, production processes and storage conditions can introduce additional chemical modifications, such as glycation or oxidation [9,10]. The co-occurrence of all these possible modifications results in a large number of so-called proteoforms for a single natural enzyme. Besides the structural complexity, these modifications might play a decisive role in the functionality of the enzyme [11], potentially influencing product performance and, therefore, they should be carefully monitored.

Suitable tools are needed to profile proteoforms present in these heterogeneous enzyme products. For this purpose, analytical workflows should be developed using separation techniques to resolve proteoforms, mass spectrometry (MS) to identify molecular entities, and activity assays to determine functionality. Moreover, it is key that the native state of the protein is maintained during separation to allow functional characterization. Therefore, native liquid chromatographic (LC) approaches should be employed instead of conventional denaturing methodologies (e.g., reversed phase LC). To date, various native protein separation techniques are available that can distinguish proteoforms based on their size, charge, hydrophobicity, or specific post-translational modifications. Unfortunately, there is not a universal native LC mode to resolve all proteoforms. As a result, a combination of orthogonal native LC modes is often required for full characterization. For instance, native size exclusion chromatography (SEC) can be employed to separate size variants of proteins (e.g., fragments and aggregates) as well as to purify samples by removal of interfering background proteins. Moreover, SEC has already found its application hyphenated with MS to characterize therapeutic proteins [12,13]. Ion exchange chromatography (IEX) has been widely established to characterize proteoforms with a difference in charge due to, e.g., deamidation, sialylation of glycans, and phosphorylation [14]. In particular, IEX methods eluting with pH gradients have recently been shown to provide robust separation of proteoforms and enable online MS coupling for identification [15,16,17,18]. Another interesting native LC approach is boronate affinity chromatography (BAC), which can be employed to selectively enrich glycated species. The separation of BAC is based on the specific and reversible interaction between the boronic ligands and cis-diol structures (i.e., glycated proteoforms) [9,19,20,21,22]. Currently, BAC is a widely applied technique to determine the glycation levels of monoclonal antibodies [23], endogenous proteins [24], and milk proteins [25]. Besides the efficient separation of these specific proteoforms, an advantage of BAC is the possibility to work at a preparative scale enabling the collection of sufficient material for further functional tests in a single run.

Here, we describe an analytical workflow combining nondenaturing chromatographic techniques, MS detection, and functional assays to establish the structure–function relationships of proteoforms of an industrially relevant endo-1,4-β-xylanase (ENDO-I). ENDO-I is produced by *Aspergillus niger* and used in baking applications to enhance bread dough development and improve crumb structure [1,4,5]. Interestingly, significant browning is observed when this enzyme is stored at high temperatures. In this accelerated stability study, we investigated the granulated enzyme product stored under different conditions, i.e., at −20 °C or a stress temperature of 40 °C for 4 and 20 weeks. To investigate in detail the changes of the product, we developed SEC, IEX, and BAC methods to resolve proteoforms based on size, charge, and presence of glycation. The activity of proteoforms was monitored during the LC method optimization, resulting in an interplay of improving the separation quality and maintaining the protein activity. Altogether, this comprehensive approach can be used as a workflow for the in-depth characterization of xylanases and enzymes in general.

## 2. Results

An industrially produced ENDO-I granulated enzyme product expressed in *Aspergillus niger* was employed as a model sample. In addition to the reference sample (stored at −20 °C), ENDO-I was kept at an elevated temperature (40 °C for 4 and 20 weeks) to evaluate the influence of temperature stress on the enzyme. Based on the sequence of ENDO-I (Figure S1), a theoretical molecular mass of 19,838.9 Da was calculated, and an isoelectric point (pI) of around 3.9 was predicted. This enzyme shows optimal activity around pH 3. All samples were structurally and functionally characterized after enrichment with different native separation techniques.

### 2.1. ENDO-I Purification Using SEC

A first inspection of the enzyme loaded on an SDS-PAGE gel showed the presence of multiple species (Figure S2). Besides a clear band at around 20 kDa of ENDO-I, we observed additional species with higher molecular weight (between 75 and 150 kDa). Therefore, prior to the structural and functional characterization of ENDO-I proteoforms, the enzyme was purified from higher molecular mass proteins in the sample. Due to the large size difference between the proteins, SEC was employed using both UV and MS detection. As functional characterization was intended, it was of utmost importance to maintain the native conformation and functionality of the enzyme during the separation. Hence, we evaluated the effect of the mobile phase composition on enzyme activity. The sample was dissolved in different mobile phases (i.e., varying in type of salt and pH) and the activity was measured (Figure S3).

Both the type of salt and the pH appeared to have a tremendous influence on the stability of the enzyme. The highest specific activity was detected using an ammonium acetate solution at pH 5.0, whilst the use of ammonium formate or ammonium bicarbonate solutions resulted in a lower specific activity. The specific activity in ammonium acetate was 78.7 U/mg protein, and a decrease was observed to 69.5 and 9.0 U/mg protein for ammonium formate and bicarbonate, respectively (Figure S3a). Furthermore, an increase of the pH of the ammonium acetate solution to pH 6.0 or 8.6 led to a complete loss of activity (Figure S3b). Therefore, we chose a mobile phase containing 100 mM ammonium acetate at pH 5.0. An isocratic method of 30 min yielded sufficient separation of the main peak of ENDO-I from other sample components.

**Figure 1:**
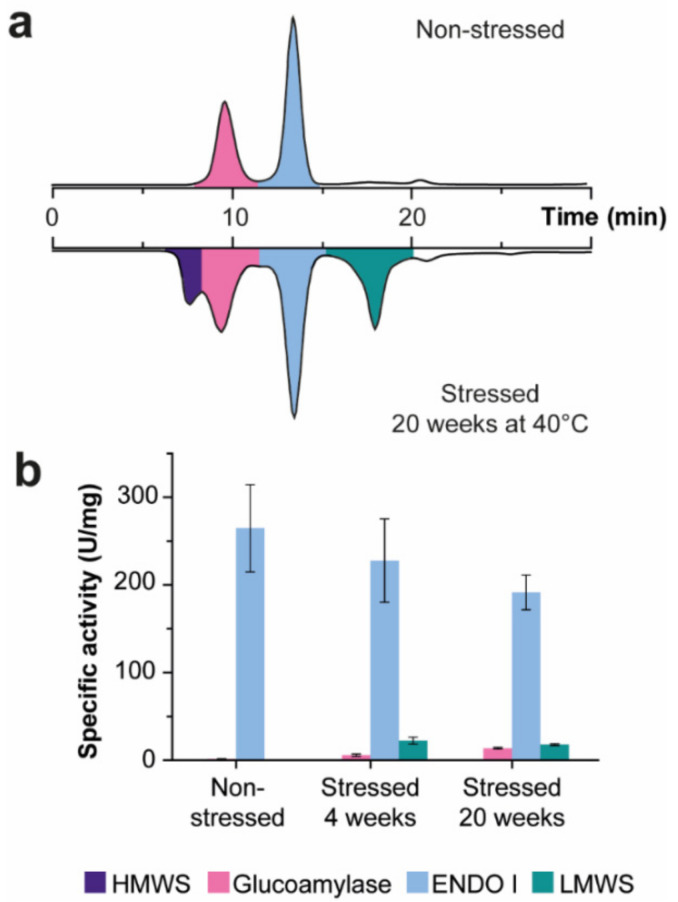
SEC analysis for the purification of ENDO-I samples. (**a**) SEC−UV chromatogram of non-stressed ENDO-I (upper part) and temperature-stressed ENDO-I for 20 weeks at 40 °C (lower part). The retention times and relative peak areas can be found in Table S2. All fractions indicated with color were collected for activity measurements. (**b**) Specific activity (U/mg protein) of the collected fractions was calculated based on the measured activity and protein concentration. The activity was measured with the XylX6 assay and the protein concentration with the BCA assay (Table S1). The measurements were performed in triplicate, and the error bars represent the standard deviation. Results of the statistical analysis of the different samples is presented in Table S3.

Figure 1a illustrates the SEC profile obtained for the non-stressed sample (upper chromatogram), showing the separation of two distinct peaks. Zero-charge deconvolution of the molecular ions at 13.3 min (indicated in blue) revealed a molecular weight of 19,838.1 Da, indicating that this peak contains the ENDO-I enzyme (theoretical mass of 19,838.9 Da). The peak eluting at 9.5 min (highlighted in pink) contained a heterogeneous protein with a higher molecular weight between 79 and 83 kDa, where the proteoforms show a mass difference of 162 Da, indicating the presence of a highly mannosylated protein (Figure S4). Fraction collection of both peaks and analysis with MALDI−ISD FT−ICR MS confirmed that the peak at 13.3 min indeed contains ENDO-I (Figure S5a). The other peak corresponds to a glucoamylase, which is co-expressed with ENDO-I during the production process (Figure S5b). Subsequently, the activity of the collected fractions was determined leading to a specific activity of 262 and 0.8 U/mg protein for ENDO-I and the glucoamylase, respectively (Figure 1b). The detected activity in the glucoamylase fraction is most likely due to insufficient fraction collection (i.e., collection of a minor part of ENDO-I). The recovery of the activity of the ENDO-I fraction compared to the start material was 81% (Table S1), which can be explained by the choice to not include the front and the tail of the peaks because of the risk of collecting other species.

Next, we analyzed the ENDO-I samples after temperature stress (i.e., 4 and 20 weeks at 40 °C). For these samples, we also observed ENDO-I (blue) and glucoamylase (pink) similar to the non-stressed sample (Figure 1a, lower chromatogram). Besides those, two additional peaks could be distinguished for the stressed material, including high molecular weight (HMW) and low molecular weight LWM species (Figure 1a, lower chromatogram). The peak area of these additional peaks increases with longer storage time at 40 °C. When comparing 4 and 20 week storage at 40 °C, an increase from 2% to 11% in relative peak area in the UV chromatogram was found for the peak at 8.4 min (HMW peak; indicated in purple) and from 16% to 23% for the peak at 17.6 min (LMW peak; indicated in green) (Table S2). Using SEC−MS, the identity of these peaks could not be determined as under the employed MS conditions no clear (protein) signals were detected in the mass spectra (Figure S6). Therefore, we investigated these fractions, both with and without an additional reduction step, using SDS−PAGE (Figure S2). The HMW peak of the non-reduced sample showed a band with a high molecular weight (>75 kDa) on SDS−PAGE. After reduction of the fraction, this band was not clearly noticeable, while a faint band appeared around the mass of ENDO-I, which could indicate that this peak contains aggregates of ENDO-I. For the HMW peak, no activity was detected. The later eluting LMW peak was not visible on SDS−PAGE, indicating that it contains probably non-proteinaceous species and/or very small fragments. Comparison of the UV chromatograms at 260 and 280 nm suggested that (part of) this peak contains non-proteinaceous species. Similar to the glucoamylase fraction, the low detected specific activity most probably is a result of traces of ENDO-I present in this fraction. Regarding the ENDO-I peak, the recovery of the activity was sufficient (i.e., 72% for 4 weeks and 65% for 20 weeks of temperature stress) since we collected only the center of the peak to minimize the presence of other species. Interestingly, the measured specific activity of the ENDO-I fraction decreased after exposure to higher temperatures to 230 and 191 U/mg protein for 4 weeks and 20 weeks at 40 °C, respectively (Figure 1b; Table S1). While the difference in specific activity between non-stressed and 4 weeks thermal stress was not yet significant, comparing the non-stressed material with 20 weeks thermal stressed sample revealed a significant difference (Table S3). The reduced activity could be caused by the presence of proteoforms containing stress-induced PTMs with lower activity. However, since SEC is not able to resolve proteoforms, other native separation modes were employed to obtain this information.

### 2.2. Charge Variant Characterization of with IEX−UV−MS

The decreased activity of the SEC-purified ENDO-I after temperature stress indicated the presence of proteoforms with altered activity. To characterize the sample in more detail, we developed an IEX method to separate proteoforms with a difference in charge. Since ENDO-I is an acidic protein (pI around 3.9), we used a strong anion exchange chromatography column (i.e., quaternary ammonium stationary phase). The mobile phases were optimized to provide good separation power and enable online MS detection while maintaining enzymatic activity. Recently, it was shown that elution with pH gradient (using volatile salts) allows good and robust separation without compromising on the quality of the MS data [16,17,18]. The start pH of the gradient was chosen to be able to retain the charged proteoforms on the column while staying within the active pH range of the enzyme leading to an optimal pH of 5.5. To achieve elution of all variants an end pH of 2.5 (or lower) was required. From the available volatile buffers, only an ammonium formate mobile phase was able to provide this pH value resulting in the choice of 50 mM formic acid (pH 2.5) as Mobile Phase B. Noteworthily, the activity of ENDO-I dissolved in ammonium formate is slightly lower compared to ammonium acetate (i.e., specific activity of ENDO-I in ammonium formate is 88% of the specific activity found after dissolving the enzyme in ammonium acetate (Figure S3a)). However, the recovered activity was considered as sufficient to investigate the difference in activity of separated proteoforms and establish their structure–function relationships.

**Figure 2:**
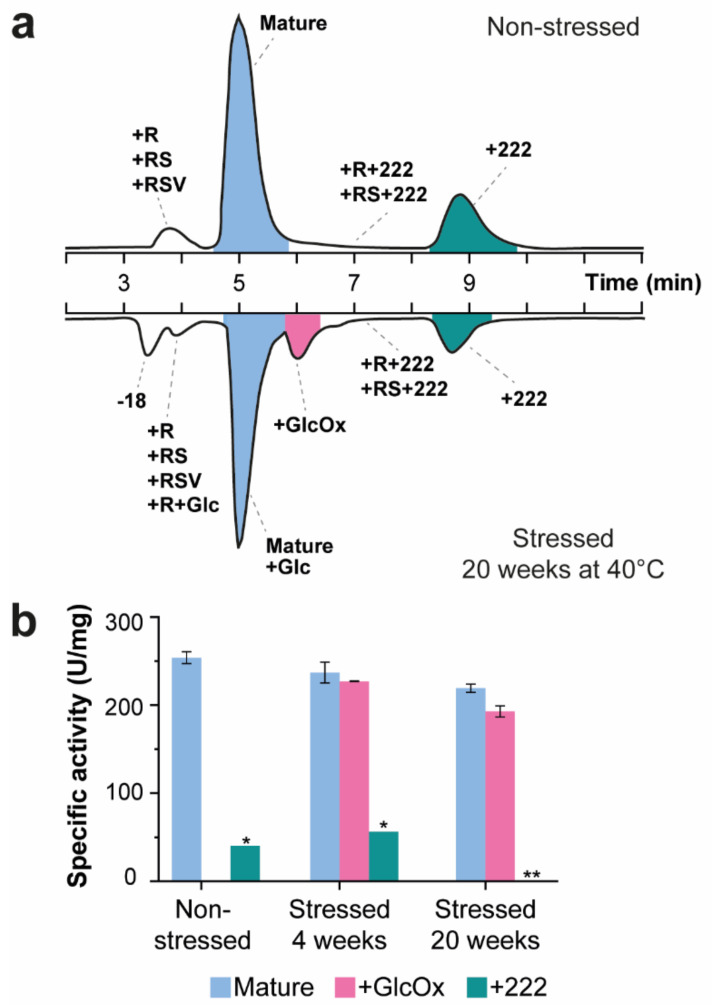
IEX−MS analysis of ENDO-I to characterize charge variants. (**a**) Base-peak chromatograms (BPCs) of the IEX separation of non-stressed (upper part) and temperature-stressed ENDO-I for 20 weeks at 40 °C (lower part). The assigned proteoforms are indicated for each peak. A complete overview of all detected masses and assignments can be found in Table S4. The peaks indicated in color were collected for further activity measurements. (**b**) The specific activity (U/mg protein) of the collected peaks was calculated from the activity and protein concentration (Table S1). The measurements were performed in triplicate and the error bars represent the standard deviation. Due to protein concentrations below the detection limit of the BCA assay, the +222 fraction of the non-stressed and the 4 weeks temperature-stressed samples were measured once (indicated with *). The protein concentration of the +222 fraction after 20 weeks temperature stress was too low to be able to calculate the specific activity (marked with **). Details on the statistical analysis of the IEX-separated samples can be found Table S3.

Analysis of SEC-purified non-stressed ENDO-I by IEX−UV−MS revealed several separated peaks (Figure 2a; Table S2). The most abundant peak eluting at 5.0 min corresponds to the mature form of ENDO-I (i.e., enzyme without signaling peptide), which contains no additional PTMs. To ensure the activity of the proteoforms after IEX, we first assessed the functionality of the mature enzyme. For the non-stressed ENDO-I, the recovery of the activity was 76% compared to the values after SEC separation, indicating that the enzyme remains active during the IEX analysis (Table S1). Besides the mature form of the enzyme, different charge variants could be detected. The peak eluting before the main peak at 3.1 min shows a mass increment of 156 Da compared to the mature form. This proteoform most probably contains an additional arginine residue (+R) at the N-terminus resulting from the incorrect processing of the signaling peptide (Figure S1). The +R variant has a shorter retention time compared to the mature enzyme due to the additional positive charge of the arginine that experiences repulsion with the positively charged stationary and thus, elutes earlier. Additionally, we detected low abundant variants with other N-terminal processing in this peak, including additional arginine and serine (+RS) or arginine, serine and valine (+RSV) variants. We observed similar activity for these species as for the mature enzyme with a specific activity of 274 U/mg protein compared to 254 U/mg protein for the mature protein (Figure 2b; Table S3).

**Figure 3:**
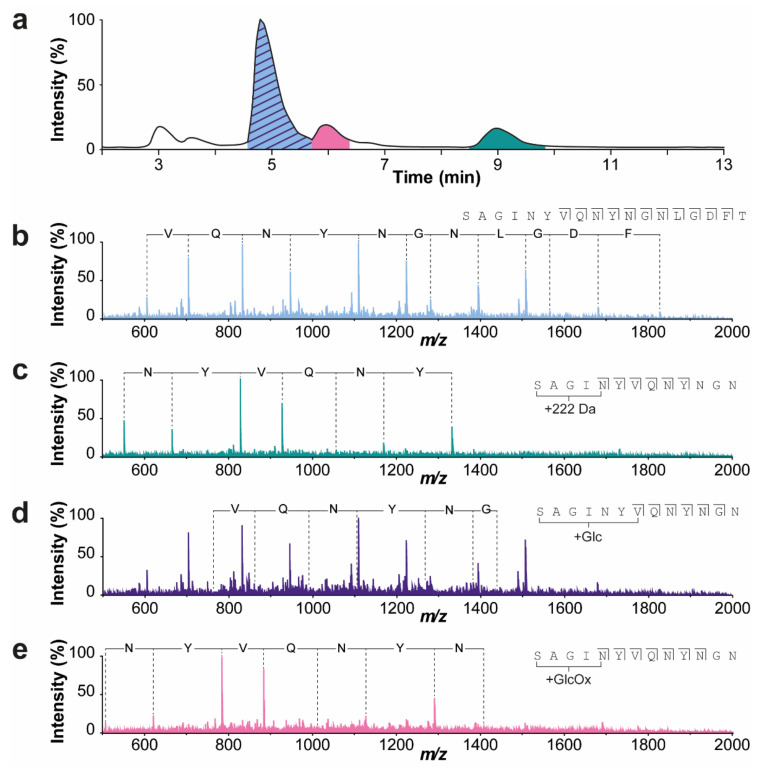
Collision-induced dissociation (CID) MS/MS spectra of the IEX peaks to determine the position of the detected modifications. (**a**) BPC of the IEX separation of the sample that was temperature-stressed for 20 weeks. (**b**) Fragmentation spectrum of the mature enzyme (displayed in blue). (**c**) Fragmentation spectrum of the +222 variant (displayed in green) eluting after the mature enzyme. (**d**) Fragmentation spectrum of the glycated variant (displayed in purple) that co-elutes together with the mature form. (**e**) Fragmentation spectrum of the glycoxidized variant (displayed in pink) eluting later than the mature peak. All these additional modifications, i.e., +222, glycated, and glycoxidized variants, are located on or in close proximity to the N-terminus. The spectra were acquired of the 8+ charge state. The isolation width and collision energy were set to 8.0 and 100.0. The exact mass differences can be found in Table S5.

The last eluting peaks (at 7.4 and 9.0 min) contain proteoforms with a mass increment of 222 Da compared to their non-modified form (i.e., mature, +R, and +RS variants). Using IEX−MS/MS, in particular collision-induced dissociation (CID), we could determine that this modification is located at (or in close proximity of) the N-terminus (Figure 3). To further investigate this modification, we collected the mature and +222 Da peak of the non-stressed sample and analyzed them with MALDI−ISD FT−ICR MS to obtain a more accurate mass. Interestingly, fragments containing this modification were only visible using negative mode ionization, indicating that this modification causes a loss of positive charge or introduces an additional negative charge. Using this approach, we could confirm that this modification is indeed situated on the N-terminus, and we acquired a mass difference of 178.0996 ± 0.0059 Da (Figure S7). When correcting for a CO_2_ loss that is considered common in negative mode MALDI−ISD FT−ICR MS, we obtain a mass difference of 222.08940 Da corresponding to a molecular formula of C_12_H_14_O_4_. However, this derived composition does not match any common N-terminal modifications, leaving us with a compositional yet no structural assignment of this proteoform. Activity measurements of this fraction showed low specific activity of 65.0 U/mg protein compared to the 254 U/mg protein for the mature enzyme peak (Figure 2b).

IEX−MS analysis of the temperature-stressed samples showed additional species compared to the non-stressed ENDO-I (Figure 2a; lower chromatogram). The mass spectra of the main peak revealed, next to ENDO-I, the presence of an early glycation product with a mass of 20,000.0 Da (i.e., attachment of glucose to the protein backbone; mass difference of +162 Da). It is known that glycation levels may be increased by exposure to higher storage temperatures [26]. Unfortunately, these species were not resolved from their non-glycated forms, but rather appeared as a shoulder of the main peak. IEX−MS/MS analysis revealed that the glycation is located on the N-terminus (Figure 3d). In addition, species with a mass of 20,016.6 Da eluting at around 6.6 min was detected. This proteoform with a mass increment of 16.6 Da compared to the glycated proteoform most probably corresponds to a glycoxidation product (i.e., early glycation products that undergo oxidative degradation [27]). Fragmentation of this peak indicated that the modification is located at the N-terminus (Figure 3e). To assess if the glycated and glycoxidized proteoforms were responsible for the activity decrease compared to the non-stressed product, these peaks were collected, and their activity was measured. The peak containing the mature form showed a slight decrease in specific activity for the temperature-stressed samples compared to the non-stressed sample, especially notable for the 20 weeks stress material (Figure 2b; Tables S1 and S3). In particular, compared to the non-stressed sample, 93% of the initial specific activity was found back after 4 weeks, which further decreased to 86% after 20 weeks of thermal stress. The decrease in specific activity was found to be significant after 20 weeks of temperature stress (Table S3). The specific activity of the glycoxidized variant was also lower compared to the mature peak of the non-stressed sample (i.e., 89% of specific activity compared to non-stressed ENDO-I for 4 weeks stress and 85% recovered compared to non-stressed ENDO-I for 20 weeks stress) (Figure 2b). The combination of an increasing level of glycation and decreasing activity suggests that these proteoforms are (partly) responsible for the reduced activity. To further investigate the effect of glycation, we employed BAC to specifically enrich for glycated proteoforms as IEX was not able to resolve these species. Finally, we also detected variants with N-terminal truncation (i.e., +R, +RS, and +RSV variants) (Figure 2a). The specific activity of this mixture of species was not significantly different from the mature ENDO-I fraction (Table S3). Additionally, the glycated form of the +R variant was detected in the stressed samples. Nevertheless, this peak overlaps with another temperature stress induced species showing a loss of 18 Da compared to the mature enzyme, which elutes at 4.4 min. Due to the co-elution of these species, we could not assess the activity of this peak containing different N-terminal variants and the glycated +R proteoform of the stressed samples. An overview of all detected masses and assignments of the proteoforms can be found in Table S4.

### 2.3. BAC for the Enrichment of Glycated Proteoforms

To obtain better insights on the effect of glycation on ENDO-I, we developed a BAC method to selectively enrich for these proteoforms. The specific interaction between glycated proteins with the boronic acid stationary phase is most stable under alkaline conditions (pH > 8.2) [23,28]. Therefore, the BAC separation is preferably performed at this pH or higher. However, we found that the activity of ENDO-I was lost after dissolving this enzyme in mobile phases at this pH (Figure S3b). Decreasing the mobile phase pH from 8.6 to 6.5 was still not sufficient to retain enzyme activity, since only 9% of the observed specific activity obtained at pH 5.0 was maintained at pH 6.5. Additionally, mobile phases with pH below 6.5 were not able to retain any glycated species. Therefore, we evaluated if the loss of ENDO-I activity was reversible by limited exposure to pH 8.6. To this end, the pH was immediately reduced after fraction collection and prior to activity measurements by the addition of acetic acid. Using this strategy, we were able to (partially) preserve the activity of ENDO-I after BAC separation at pH 8.6, i.e., 79% of the specific activity measured with ammonium acetate (pH 5.0) was recovered (Figure S3b). As the specific interactions are most stable, this pH and the activity could be maintained, and pH 8.6 was selected for ENDO-I separation.

**Figure 4:**
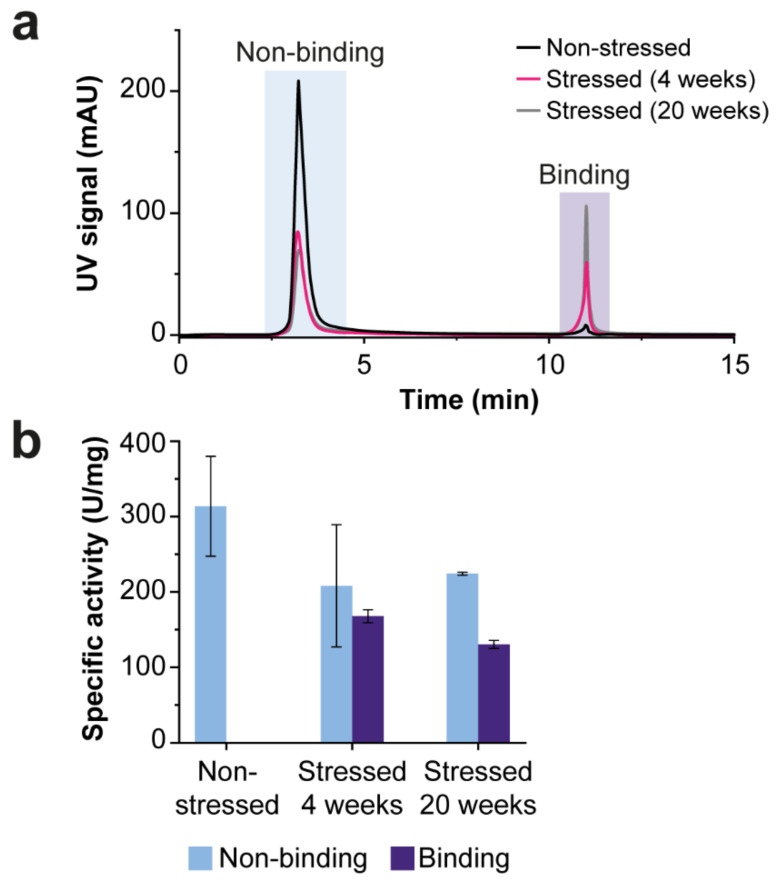
BAC separation of glycated and non-glycated proteoforms of ENDO-I. (**a**) The overlay of the UV chromatograms (acquired at 280 nm) of the SEC-purified samples measured with the optimized BAC method. The non-stressed sample trace is black, and the temperature-stressed enzyme traces are pink (4 weeks) and gray (20 weeks). The peak areas of the non-binding and binding peak can be found in Table S3. (**b**) Specific activity of the non-binding (blue) and binding (purple) peak of the BAC separations. The measured activity and protein concentration are reported in Table S1. The measurements were performed in triplicate, and the error bars represent the standard deviation. Additional information on the statistical analysis of the BAC-separated samples can be found Table S3.

Besides the alkaline pH, the salt concentration in mobile phases can significantly influence the separation quality. Often, a buffer containing 250 mM ammonium acetate is employed [29,30]. However, when using this concentration, we observed a high relative area of the binding peak for the non-stressed sample. Since the non-stressed sample should contain no to minor glycation, this peak is most likely due to nonspecific secondary interactions that take place between the proteins and the stationary phase. For this reason, we further optimized the Mobile Phase By examining the effect of different ammonium acetate concentrations on the relative peak areas. For both the stressed and non-stressed samples, we observed that the binding peak area decreased with decreasing ammonium acetate concentration (Figure S8a,b; Table S6). Moreover, ionic strengths below 100 mM resulted in not-repeatable separations. Further reduction of secondary interactions is often accomplished by using shielding agents (e.g., Tris) and/or additional salts (e.g., NaCl or MgCl_2_) [31]. However, we observed the opposite effect, i.e., the binding peak of the non-stressed ENDO-I increased after the addition of 50 mM MgCl_2_ to the mobile phase (Figure S8c,d; Table S6). Furthermore, the effect of the sorbitol concentration was investigated for the thermal-stressed ENDO-I, where the non-binding peak remains mostly constant at all sorbitol concentrations, a decrease in the peak area of the binding peak was observed when eluting with sorbitol concentrations below 400 mM due to incomplete elution of the glycated proteoforms (Figure S8e; Table S6). Finally, we reduced the gradient time as much as possible to ensure the lowest exposure time of ENDO-I to the high pH mobile phase. In particular, we flushed the column 5 min with Mobile Phase A to promote binding followed by an elution step of 10 min. In summary, a binding buffer containing 100 mM ammonium acetate at pH 8.6 minimized the interference of secondary interactions and an elution buffer with 100 mM ammonium acetate, and 400 mM sorbitol (pH 8.6) allowed elution of the glycated species (Figure 4a).

Using this optimized method, the majority of proteoforms of the non-stressed ENDO-I eluted in the non-binding peak with only a minor binding peak with a relative area of 4% (Table S2). For the temperature-stressed materials, the relative area of the binding peak was higher than the non-stressed sample, and it was increasing with exposure time to 40 °C (i.e., from 28% for 4 weeks and 41% for 20 weeks). To obtain more information on the presence of glycated variants in the non-binding and binding peaks, both peaks of the temperature-stressed ENDO-I (20 weeks) were collected, concentrated, and measured with SEC-MS. In this way, we could confirm that the non-binding peak contains only a minor amount of glycated proteoforms (Figure S9). Additionally, we noticed that the binding peak contained, besides glycated and glycoxidized proteoforms, the –18 Da variant. Since this species is not glycated, it probably experiences unspecific interaction with the boronate affinity column surface.

For further functional assessment, the specific activity of the non-binding and binding peak of all samples was determined (Figure 4b). In first instance, we expected to find similar specific activities for the non-binding peaks of all samples. However, a decrease in specific activity was observed for the stressed samples compared to the non-stressed sample. As most of the stress-related modifications elute in the binding peak (Figure S9), the activity of the species was likely affected by the fraction collection approach and/or the storage conditions. The binding peak of the 20 week stressed material shows significantly lower specific activities compared to the non-binding peak (Table S3). This suggested that the altered activity is (in part) due to glycation of the ENDO-I.

## 3. Discussion

A complete analytical workflow for the structural and functional characterization of an industrially produced endo-xylanase (ENDO-I) was presented employing a combination of native chromatographic techniques, MS detection, and activity assays. Using this platform, various proteoforms of ENDO-I were identified, including N-terminal processing variants. Additionally, we could monitor the influence of storage under stress temperature (40 °C) for long periods on the proteoform profile. The use of different native LC methodologies combined with MS has already been reported for the structural characterization of proteins (mainly monoclonal antibodies) and has been demonstrated to be very powerful for monitoring changes on their higher-order structure [32,33,34]. To enable the analysis of higher-order structures, the separation conditions should maintain proteins in their “native form” and therefore should not induce any changes in protein folding or induce the formation of additional proteoforms. The term native refers here to the unaltered, biologically functional states of proteins and non-covalent protein assemblies in solution under near-physiological conditions [35]. However, in our study, we consider the enzyme native when the total of recovered activity of the separated proteoforms similar compared to the non-separated enzyme. To this end, we highlight the effect of (pseudo-)native conditions on enzyme functionality, which is essential for drawing valid conclusions from these workflows on proteoform-specific activities.

To enable the determination of protein activity after separation, ideally, the native LC conditions must not influence the enzymatic activity. Nevertheless, often the required separation buffers are not able to (fully) maintain the enzyme activity. In those cases, additional post-separation strategies (e.g., change of buffer salt and/or pH prior to activity measurements) should be applied to restore the enzyme activity and enable functional assessment. To explore the effect of common (MS-compatible) separation conditions on ENDO-I activity, we monitored the enzyme activity in different ammonium buffers (acetate, formate, and bicarbonate), showing that ammonium acetate was most suitable for preservation of ENDO-I activity. Ammonium formate resulted in a slightly reduced activity at pH 5.0, while ammonium bicarbonate tremendously decreased the activity of ENDO-I. These results are in line with Ventouri et al. [36], where it was shown that ammonium acetate preserved the protein structure (e.g., for myoglobin, ovalbumin, and thyroglobulin), whereas ammonium formate and bicarbonate salts induced (partial) denaturation of proteins. Therefore, we aimed to use ammonium acetate-based mobile phases where possible. However, ammonium acetate buffers were not able to elute the proteoforms of ENDO-I from the IEX column. In this case, a compromise between enzyme activity and separation requirements was necessary. The pH of the Mobile Phase Also influences enzyme activity. ENDO-I is active and fairly stable between 1.5 and 6. However, BAC has limited flexibility in terms of mobile phase pH due to its characteristic separation mechanism. In particular, the BAC separation should be performed at pH values above 8.2, where ENDO-I is not stable. Therefore, we used a strategy, the pH is immediately decreased after separation, to reverse the loss of activity due to high pH. Finally, the risk of enzyme inactivation can be minimized by using short separation times making short gradients desirable.

For the complete characterization of proteins, a platform using multiple complementary separation techniques is often required, as each separation technique provides unique information on specific variants and/or modifications [37]. For instance, SEC can purify the enzyme of interest from other proteins or size variants present in the sample, but proteoforms resulting in a smaller difference in size can often not be separated [12]. In particular, we could detect a decrease in specific activity of the SEC-separated ENDO-I peak after temperature stress. However, the exact change in proteoform profile responsible for this decrease remained unknown. In this case, orthogonal separation techniques, such as IEX or BAC, are essential. IEX is a suitable technique for the separation of charge variants (e.g., N-terminal variants and glycoxidized variants), whereas glycated proteoforms co-elute with their non-glycated variants [34,38]. To isolate these glycated proteoforms, it was necessary to use the selectively separation of BAC. This novel analytical workflow has proven to be useful for the establishment of structure–function relationships of ENDO-I proteoforms. We have shown that ENDO-I exists of a mixture of proteoforms. Next to the major active mature form, some low-abundance variants show differences in activity. Whereas the variants with altered N-termini showed similar activity as the mature enzyme, for the +222 Da modification, low-to-no activity was detected. In addition, using this approach, we could monitor low abundant stress-induced modifications, revealing the presence of glycation and glycoxidation, which both resulted in lowered activity compared to the mature enzyme.

Altogether, the combination of orthogonal separation techniques generates deep insights into a complex industrial enzyme sample, herewith supporting the development of improved enzymes. Overall, the developed workflow provided a comprehensive overview of all structural features and enabled the study of their impact on function. We foresee that the reported strategy can be extended to other industrial enzymes by tailoring the separation conditions based on the specific enzyme characteristics.

## 4. Materials and Methods

### 4.1. Materials

Ammonium acetate (≥98%), acetic acid (≥99%), bovine serum albumin (BSA) (≥98%), D-Sorbitol (≥99%), 1,5-diaminonaphthalene (1,5-DAN) (97%), magnesium chloride (≥98%), and tris(hydroxymethyl)aminomethane (≥99.8%) were purchased from Sigma (Steinheim, Germany). Ammonium formate (>97.0%) and ammonium bicarbonate (≥99.5%) were obtained from Honeywell Fluka (Steinheim, Germany). Formic acid (FA) (>98%) was purchased from Riedel-De Haen (Seelze, Germany). 2-mercaptoethanol was purchased from Amresco (Solon, OH, USA). Sodium hydroxide was obtained from Merck Millipore (Darmstadt, Germany). Acetonitrile (ACN) and methanol (MeOH) were purchased from Actu-All chemicals (Randtmeer, the Netherlands). Deionized water (milli-Q) was obtained from a Purelab ultra system (ELGA Labwater, Ede, the Netherlands). Samples of granulated *Aspergillus niger* endo-1,4-β-xylanase I (EC 3.2.1.8) stored under different conditions were provided by DSM (Delft, the Netherlands). These samples were stored at −20 °C until use.

### 4.2. Size Exclusion Chromatography–Mass Spectrometry

For preparative SEC measurements, an Äkta pure instrument (Cytiva, Freiburg im Breisgau, Germany) was used with a Superdex column (10 × 300 mm, 13 µm) from GE healthcare (Uppsala, Sweden). An isocratic run of 30 min was performed using a mobile phase comprised of 100 mM ammonium acetate at pH 5.0. The pH of the solution was adjusted using acetic acid. Prior to injection of ENDO-I, a gel filtration standard (Bio-Rad Laboratories Inc., Hercules, CA, USA) was analyzed. The injected amount of ENDO-I was 25 mg, and UV chromatograms were recorded at 280 nm. Fractions were collected every 0.5 min, and fractions of the same peak were combined. The ENDO-I fractions have an estimated concentration of around 7.0 mg/mL based on the relative peak area in the UV chromatogram. This fraction was used for further IEX and BAC analyses.

SEC−MS measurements were performed using a biocompatible Ultimate 3000 instrument (Thermo Fisher Scientific, Landsmeer, The Netherlands) containing a quaternary pump, autosampler, column thermostat, variable wavelength detector, and pH and conductivity monitor. A TSKgel UP-SW3000 (4.6 × 150 mm, 2 µm) was purchased from TOSOH (Griesheim, Germany). The same Mobile Phase As for the preparative SEC was used. The analysis time was 20 min, the flow rate was 0.25 mL/min, and the injected amount was 30 µg. An Impact qTOF-MS (Bruker Daltonics, Bremen, Germany) was used in positive-ion mode and, prior to ionization, the flow rate was reduced via a post-separation flow splitter (1:5). The capillary voltage was set to 3700 V, nebulizer gas to 0.8 bar, dry gas flow to 5 L/min, and dry temperature to 200 °C. Funnel 1 was set to 190 Vpp, Funnel 2 to 600 Vpp, and hexapole to 210 Vpp. The quadrupole ion energy and collision cell voltage were operated at 5.0 and 10.0 eV, respectively. The collision cell RF was 1500 Vpp. The transfer and pre-pulse storage times were set to 100.0 and 30.0 µs. For in-source collision-induced dissociation, 150 eV was used. Spectra were acquired in *m*/*z* range between 600–7000. Deconvolution of the mass spectra was performed using the maximum entropy algorithm in the DataAnalysis software from Bruker Daltonics (version 5, Bremen, Germany)

### 4.3. Gel Electrophoresis

The SEC fractions of stressed ENDO-I (20 weeks at 40 °C) were loaded on SDS-PAGE. To obtain more information on the nature of the SEC-separated species, the fractions were both analyzed without additional sample preparation (non-reducing conditions) and after reduction of the disulfide bridges (reducing conditions). This reduction was performed by the addition of mercapoethanol to the LDS sample buffer (Invitrogen, Thermo Fischer Scientific, Carlsbad, CA, USA). For SEC fractions 1, 2, and 4, 6 µL LDS buffer was added to the 18 µL sample. For the nonseparated ENDO-I sample, the 5 µL sample was mixed with 13 µL milli-Q and 6 µL LDS buffer. For SEC fraction 3, 10 µL was added to 5 µL milli-Q and 6 µL LDS buffer. The samples were incubated at 70 °C for 10 min (nonreducing conditions) or at 95 °C for 10 min (reducing conditions). From the samples, 20 µL was loaded on the gels, and 5 µL was loaded of the Precision Plus Protein molecular weight ladder (Bio-Rad Laboratories Inc., Hercules, CA, USA). The SDS−PAGE gels (NuPage 4–12% Bis-Tris) were run at 200 V for 37 min followed by washing with milli-Q and staining with SimplyBlue SafeStain (Invitrogen, Thermo Fischer Scientific, Carlsbad, CA, USA).

### 4.4. Ion Exchange Chromatography–Mass Spectrometry

The IEX separation was performed using the same system as for SEC−MS. For the separation, a ProPac SAX-10 column (2.0 × 250 mm, 10 µm) was used (Thermo Fisher Scientific, Landsmeer, The Netherlands). The optimal mobile phases were 50 mM ammonium formate at pH 5.5 (A) and 50 mM formic acid at pH 2.5 (B). The programmed gradient increased from 30% B to 75% B in 0.1 min followed by an increase to 78% B in 15 min. Thereafter, a cleaning (100% B for 5 min) and equilibration step (30% B for 20 min) were performed prior to the injection of the next sample. The flow rate, column temperature, and UV wavelength were 0.25 mL/min, 25 °C, and 280 nm, respectively. The injected amount was 20 µg (UV detection), 30 µg (MS detection), or 180 µg (fraction collection). The IEX−MS measurements were performed using the same MS system and setting as SEC−MS. For the IEX−MS/MS measurements, CID fragmentation was performed. The isolation mass and width were set to 2508.60 and 8.0, respectively. The collision energy and isCID energy were 100.0 and 0.0 V, respectively.

### 4.5. Boronate Affinity Chromatography

BAC separations were performed on an Agilent HPLC 1100 system (Waldbronn, Germany) equipped with autosampler, quaternary pump, column thermostat, and diode array detector. A TSKgel Boronate-5PW column was used (7.5 × 75 mm, 10 µm) from Tosoh Bioscience (Montgomeryville, PA, USA). Mobile Phase A consisted of 100 mM ammonium acetate and Mobile Phase B of 100 mM ammonium acetate with 400 mM sorbitol, both at pH 8.6. The pH of the mobile phases was adjusted with 50% NaOH. For the separation, a step-gradient was employed. First the column was flushed with 100% Mobile Phase A for 6 min, followed by flushing with 100% Mobile Phase B for 10 min. Thereafter, the column was cleaned with 100 mM acetic acid for 5 min. Finally, a 5 min equilibration step was performed with Mobile Phase A. The column temperature, flow rate, and UV wavelength were set to 40 °C, 0.8 mL/min, and 280 nm, respectively. A blank injection was made with each sequence prior to sample injections. For determination of the glycation level in the binding and non-binding peak, 36 µg (20 µL) ENDO-I sample was injected, and fractions were collected and concentrated using Vivaspin 2 spin filters with 3 kDa cutoff (GE Healthcare, Munich, Germany). Thereafter, the fractions were measured with SEC−MS.

### 4.6. MALDI Insource Decay Mass Spectrometry Measurements

The MALDI−insource decay (ISD) FT−ICR MS was performed as previously reported [39,40,41]. In short, the SEC fractions (ENDO-I and glucoamylase) and IEX fractions (mature and +222 Da peak) were collected, and buffer exchanged using 3 kDa Vivaspin 2 filters to 5% MeOH or 50 mM ammonium formate, respectively. From these samples, 2 μL was spotted onto a polished steel MALDI target plate. Subsequently, 1 μL of 1,5-DAN matrix (saturated solution of 1,5-DAN prepared in solution of 50% acetonitrile, 49.95% milli-Q and 0.05% FA) was added and mixed gently until the onset of small crystal formation. The sample was immediately measured after drying to air at room temperature using a 15 T solariX XR FT-ICR mass spectrometer (Bruker Daltonics, Bremen, Germany) equipped with a CombiSource and a ParaCell. The system was operated in (positive or negative) MALDI-mode using a Smartbeam-II Laser System (Bruker Daltonics) at a frequency of 500 Hz with 200 laser shots per measurement. The mass spectra were obtained using two different acquisition methods optimized in *m/z*-ranges 500–7000 and 2000–30,000 for measurements in positive mode and in *m/z*-ranges 500–5000 for measurements in negative mode.

### 4.7. Activity Measurements

The endo-xylanase assay kit from Megazyme (Wicklow, Ireland) was used to determine the activity of ENDO-I granulates and collected fractions. The XylX6 reagent was prepared by the addition of 5 mL milli-Q to the lyophilized powder, after dissolving the reagent was further diluted with 4 mL assay buffer (6.7 g/L malic acid and 2.9 g/L sodium chloride adjusted to pH 5.2 with 4 M NaOH solution). To stop the reaction, a solution of 2% (*w*/*v*) Tris (pH 10.0) was used. Five concentrations of an ENDO-I standard preparation in range 0.1–1.0 U/mL were measured with the assay to obtain a calibration line. This enzyme standard supplied by DSM was calibrated by using a viscosimetric method. The activity is expressed in U/mL, where one unit corresponds to an enzyme activity that creates a change in viscosity with a constant rate of 12.76 per min using a Ubbelohde viscosimeter while incubating the enzyme with 0.5% wheat arabinoxylan (Megazyme, P-WAX) in 0.2 M glycine/HCl buffer pH 2.75 and 47 °C. All samples were diluted in assay buffer to approximately 0.5 U/mL. For the activity measurements, 72 µL of XylX6 reagent and the diluted ENDO-I samples were separately pre-incubated at 37 °C for 3 min. Subsequently, 8 µL of ENDO-I solution was added to the XylX6 solution, and the mixture was incubated at 37 °C for 5 min. After incubation, 60 µL stop reagent was added, and the absorbance of the samples was measured at 405 nm against milli-Q. For each sample, a sample blank was measured by reversing the order of the addition of sample and stop reagent. A calibration line was prepared by plotting the delta absorbance (sample–sample blank) at a wavelength of 405 nm versus the known activity of the diluted enzymatic standard solutions (S1–S5). This calibration line was fitted according to a linear function, and the slope was determined. The amount of activity in the unknown samples was calculated as follow: Activity in U/mL = (Abs sample − Abs blank)/a × df (a = slope calibration line, df = dilution factor).

The protein concentration was determined with BCA protein assay reagents from Sigma: bicinchoninic acid solution Sigma B-9643 (Reagent A) and copper (II) sulphate.5 H_2_O, 4% (*w*/*v*) solution Sigma C-2284 (Reagent B). A calibration line was made using BSA solutions (Sigma P-5369) with final concentrations of approximately 0, 0.1, 0.25, 0.4, 0.6, 0.7, 0.85, and 1.0 mg/mL. The working reagent was prepared by mixing 50 parts Reagent A with one part Reagent B (*v*/*v*). 30 µL of an appropriately diluted ENDO-I solution was added to 180 µL working reagent and the samples were incubated at 37 °C for 30 min. The absorbance was measured at 562 nm against milli-Q. The specific activity was calculated using the measured activity divided by the protein concentration determined with the BCA assay.

The specific activity was calculated using the measured activity and protein concentration in U/mg protein (U/mL × 1/*p* (*p* = mg protein/mL)). For the statistical analysis of the fraction activities, first, the average specific activity of each fraction was calculated from the three individual measurements. Thereafter, a *t*-test test for a significant difference between two independent samples was performed.

## Supplementary Materials

The following are available online at https://www.mdpi.com/article/10.3390/ijms23031307/s1.
