# Supplementary material for: Native Liquid Chromatography and Mass Spectrometry to Structurally and Functionally Characterize Endo-Xylanase Proteoforms
Source: Int J Mol Sci. 2022 Jan 24;23(3):1307. doi: 10.3390/ijms23031307 (PMC8835838; doi:10.3390/ijms23031307)
Supplement: Supplementary file 1 [file ijms-23-01307-s001.zip › ijms-1548088-supplementary.pdf]

Supplementary information

Native liquid chromatography and mass spectrometry to structurally and functionally characterize endo-xylanase proteoforms

Guusje van Schaick [1*], Nadi el Hajjouti [1], Simone Nicolardi [1], Joost den Hartog [2], Romana Jansen [2], Rob van der Hoeven [2], Wim Bijleveld [2], Nicolas Abello [2], Manfred Wuhrer [1], Maurien Olsthoorn [2], and Elena Domínguez-Vega [1]

[1] Leiden University Medical Center, Center for Proteomics and Metabolomics, Leiden, the Netherlands
[2] DSM, Center for Analytical Innovation, Delft, the Netherlands

[*] Corresponding author

Guusje van Schaick

Leiden University Medical Center, Center for Proteomics and Metabolomics, Albinusdreef 2, 2333 ZA Leiden, the Netherlands. E-mail: g.van_schaick@lumc.nl

Table of contents

Figure S1. Sequence ENDO-I 3

Figure S2. Analysis of ENDO-I using SEC-UV and gel-based approaches 4

Figure S3. Activity of the non-separated ENDO-I 5

Figure S4. SEC-MS of non-stressed ENDO-I 6

Figure S5. MALDI-ISD-FT-ICR-MS spectrum of SEC fractions 7

Figure S6. SEC-MS of temperature-stressed ENDO-I 8

Figure S7. MALDI-ISD-FT-ICR-MS of IEX fractions 9

Figure S8. BAC method optimization 10

Figure S9. MS analysis of BAC fractions 11

Table S1. Specific activity and recovery of the ENDO-I samples after separation 12

Table S2. Retention times and relative peak area of the native separation methods 14

Table S3. Statistical analysis of specific activity differences between samples 15

Table S4. Assignment of proteoforms separated with IEX-MS 16

Table S5. Exact mass difference IEX-separated modifications 17

Table S6. BAC method optimization 18

**M K V T A A F A G L**	**L V T A F A A P A P**	**E P D L V S R** S A G	I N Y V Q N Y N G N
L G D F T Y D E S A	G T F S M Y W E D G	V S S D F V V G L G	W T T G S S N A I T
Y S A E Y S A S G S	A S Y L A V Y G W V	N Y P Q A E Y Y I V	E D Y G D Y N P **C** S
S A T S L G T V Y S	D G S T Y Q V **C** T D	T R T N E P S I T G	T S T F T Q Y F S V
R E S T R T S G T V	T V A N H F N F W A	Q H G F G N S D F N	Y Q V V A V E A W S
G A G S A S V T I S	S		

Figure S1. The sequence of endo-1,4-β-xylanase I (EC 3.2.1.8) from *Aspergillus niger* abbreviated further as ENDO-I with in purple the signal peptide, in green N-terminal truncation, and in blue the cysteines that form a disulfide bridge. The sequence contains no consensus N-glycosylation site (N-X-S/T).

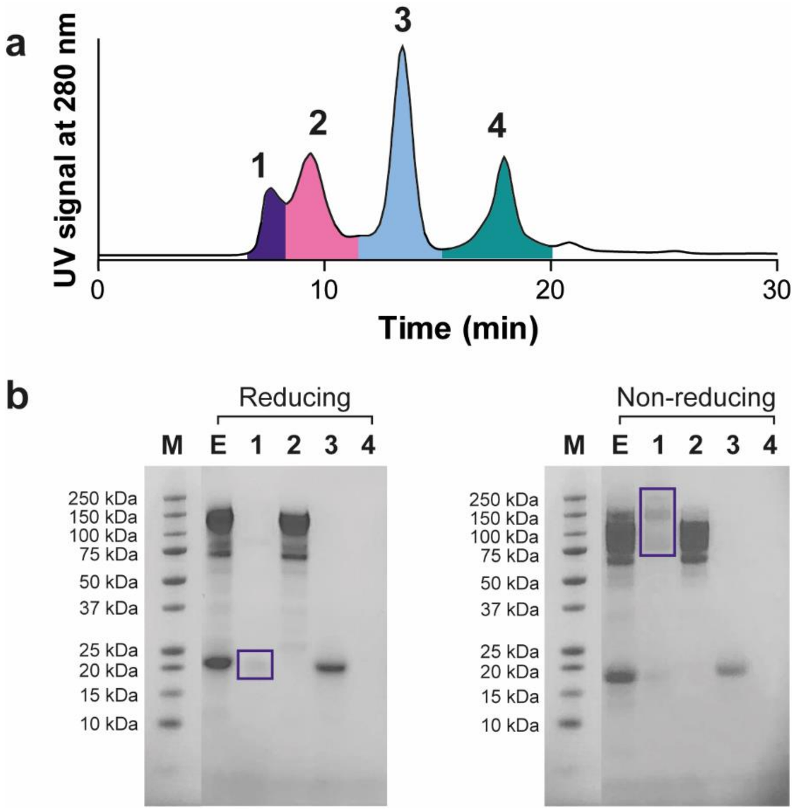

Figure S2. (**a**) SEC-UV chromatogram of temperature-stressed ENDO-I (20 weeks at 40 °C) recorded at 280 nm. (**b**) SDS-PAGE gel of reduced and non-reduced ENDO-I samples, where M is the molecular weight marker, E is the non-separated ENDO-I, and 1-4 correspond to the numbered fractions in the SEC chromatogram. The corresponding masses of the molecular weight marker are indicated on the left. Moreover, the band of fraction 1 (HWMS) is highlighted in purple for both the reducing and non-reducing gel. The non-separated ENDO-I was dissolved in 100 mM ammonium actetate (pH 5.0).

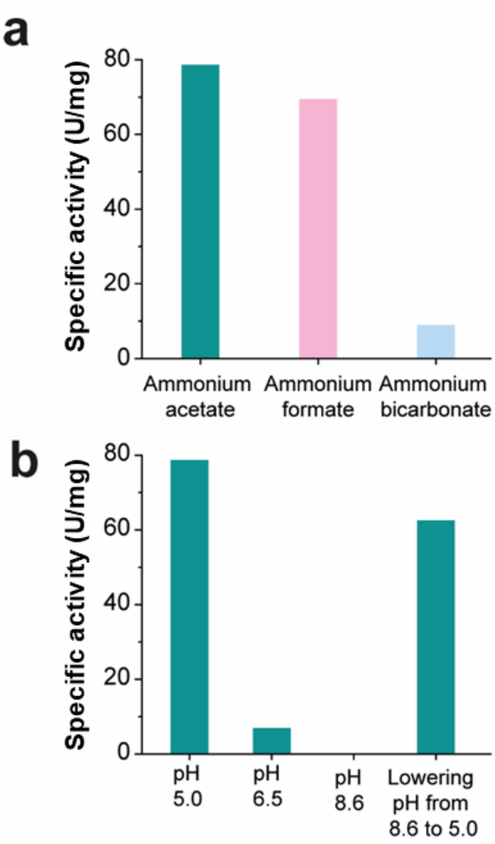

Figure S3. The specific activity (in U/mg protein) of the non-stressed ENDO-I without separation dissolved in mobile phases containing different volatile salts at various pH values. (**a**) Comparison of the specific activity after dissolving the enzyme in 100 mM ammonium acetate, formate, and bicarbonate at pH 5.0. (**b**) Specific activity of ENDO-I dissolved in 100 mM ammonium acetate at different pH values, including pH 5.0, 6.5, and 8.6. Since BAC requires separation at pH 8.6 while ENDO-I is not active anymore at this pH, immediate lowering of the pH after separation was performed. In this way, most of the activity could be preserved. *The* activity was measured with the XYLX6 assay and the protein concentration was determined using a BCA assay.

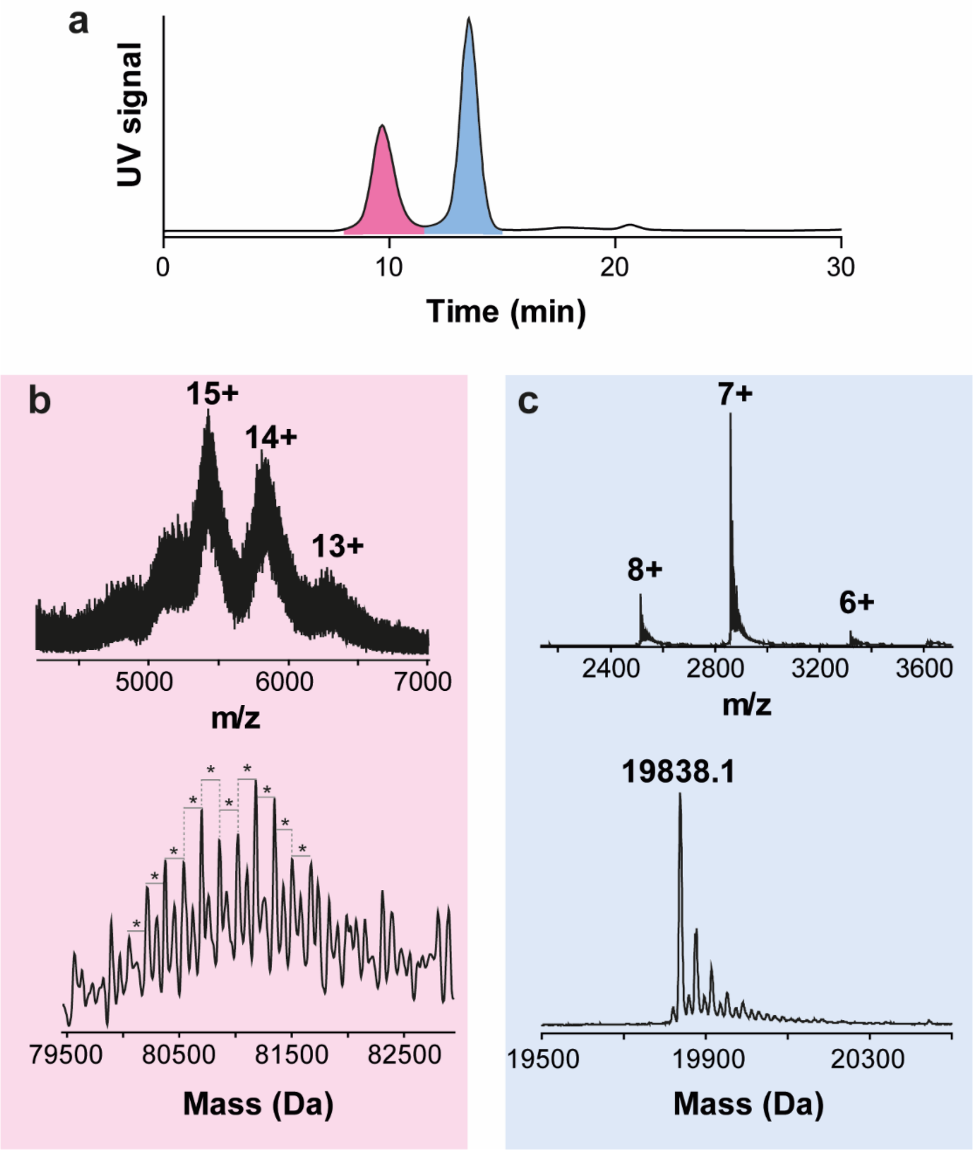

Figure S4. SEC-UV-MS measurement of non-stressed ENDO-I. (**a**) The UV chromatogram acquired at 280 nm, where the glucoamylase is indicated in pink and the ENDO-I in blue. (**b**) Mass spectrum and zero-charge deconvoluted spectrum of the glucoamylase. The mass difference indicated with * corresponds to 162 Da. (**c**) Mass spectrum and zero-charge deconvoluted spectrum of the ENDO-I peak. For both mass spectra the charge states are indicated.

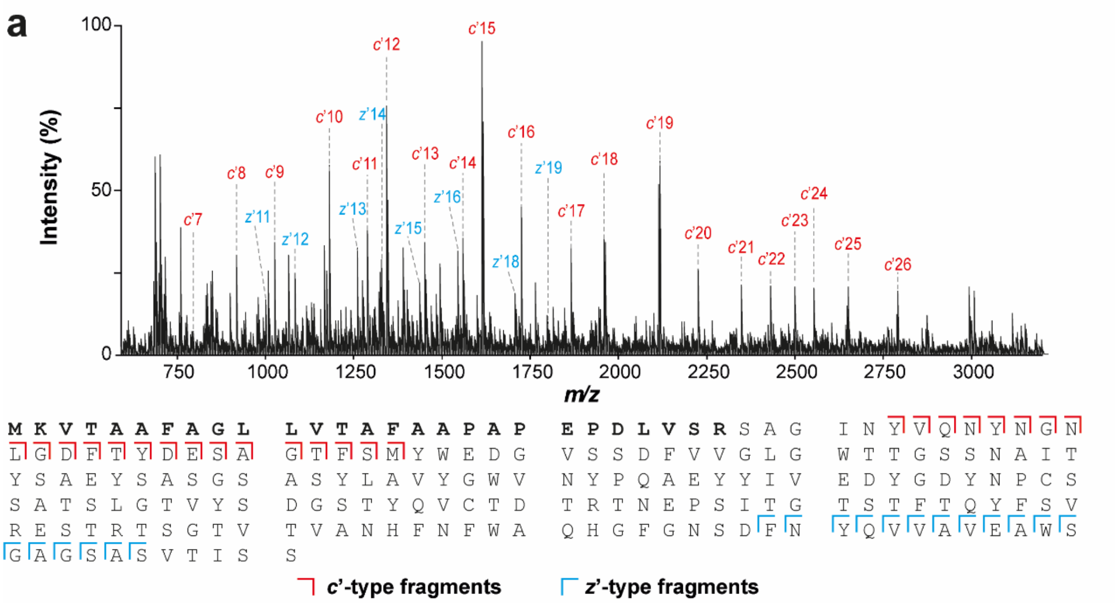

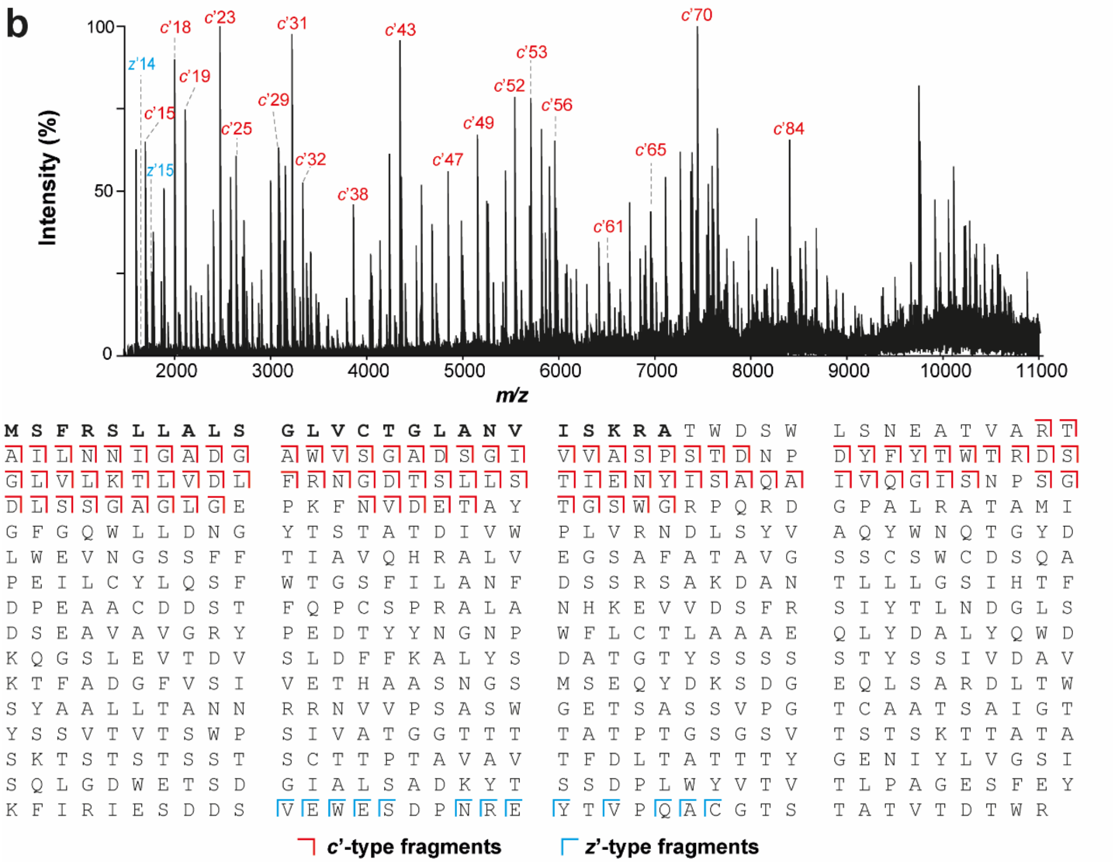

Figure S5. Positive mode MALDI-in-source decay (ISD) FT-ICR mass spectrum of ENDO-I (**a**) and glucoamylase (**b**). For both samples, high abundant *c*'-type and *z*'-type fragment ions are assigned in the spectra and the complete sequence coverage is indicated below the spectrum. The fragment ions were detected as $[M+H]^+$. For these measurements, the ENDO-I and glucoamylase peaks of SEC separation, displayed in **Figure S2,** were collected and concentrated. Subsequently, these samples were spotted on a MALDI plate and measured.

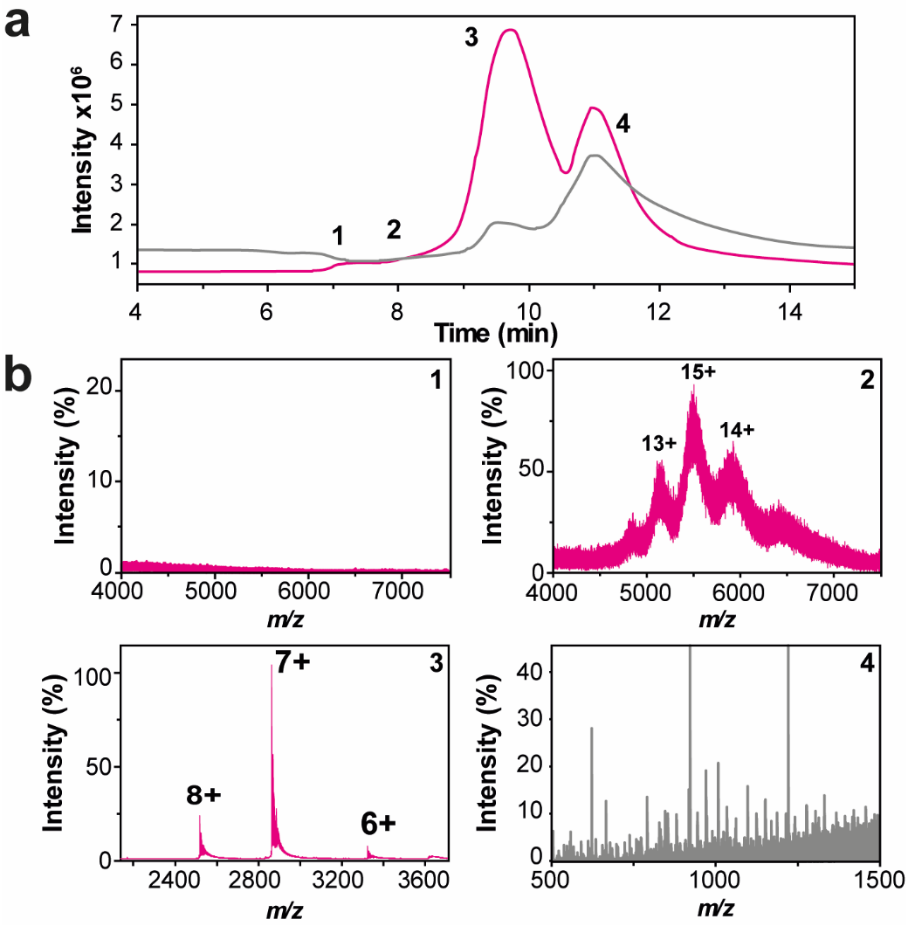

Figure S6. SEC-MS analysis of the temperature-stressed ENDO-I (20 weeks). (**a**) Overlay of the base-peak chromatograms (BPCs) of ENDO-I measured with MS method optimized for high molecular weight proteins (pink trace) and ENDO-I measured with an MS method for peptides (gray trace). (**b**) Mass spectra of the numbered peaks, where 1 is the HMW peak, 2 is the glucoamylase, 3 is ENDO-I, and 4 is the LMW peak. The color of the spectrum corresponds to the used chromatogram.

a

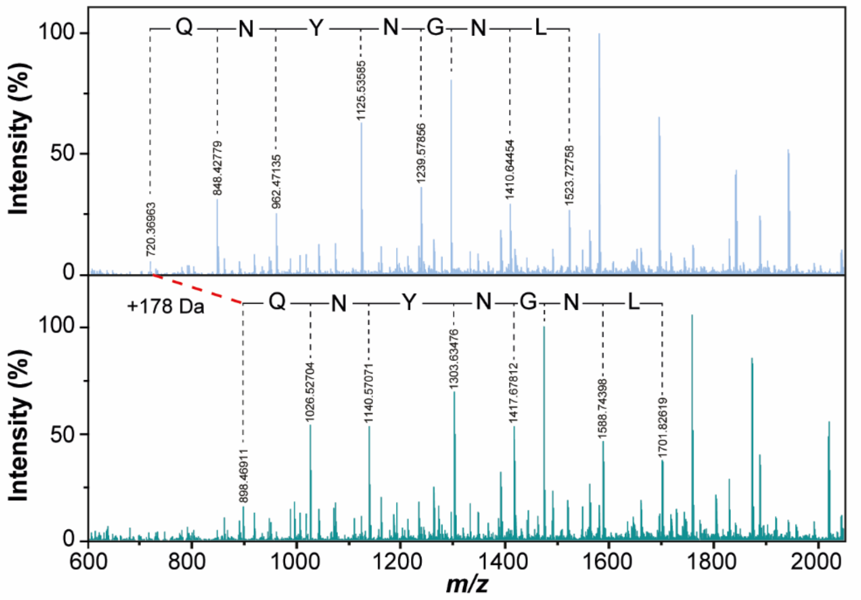

b

Fragment	*m/z* mature	*m/z* +222 Da	Difference
SAGINYV	720.3684	898.4691	178.1007
SAGINYVQ	848.4275	1026.5270	178.0995
SAGINYVQN	962.4710	1140.5707	178.0998
SAGINYVQNY	1125.5354	1303.6348	178.0994
SAGINYVQNYN	1239.5787	1417.6781	178.0995
SAGINYVQNYNG	1296.6002	1474.6998	178.0997
SAGINYVQNYNGN	1410.6445	1588.7440	178.0994
SAGINYVQNYNGNL	1523.7276	1701.8262	178.0986

Average	178.0996
SD	0.0006

Figure S7. (**a**) Negative mode MALDI-ISD FT-ICR mass spectrum of IEX-separated mature ENDO-I (upper spectrum) and ENDO-I modified with +222 Da (lower spectrum). (**b**) Overview of the measured *m/z* values of the mature protein and +222 variant for different *c′*-type fragment ions. The mass difference calculated for all fragments is shown, including the average and standard deviation.

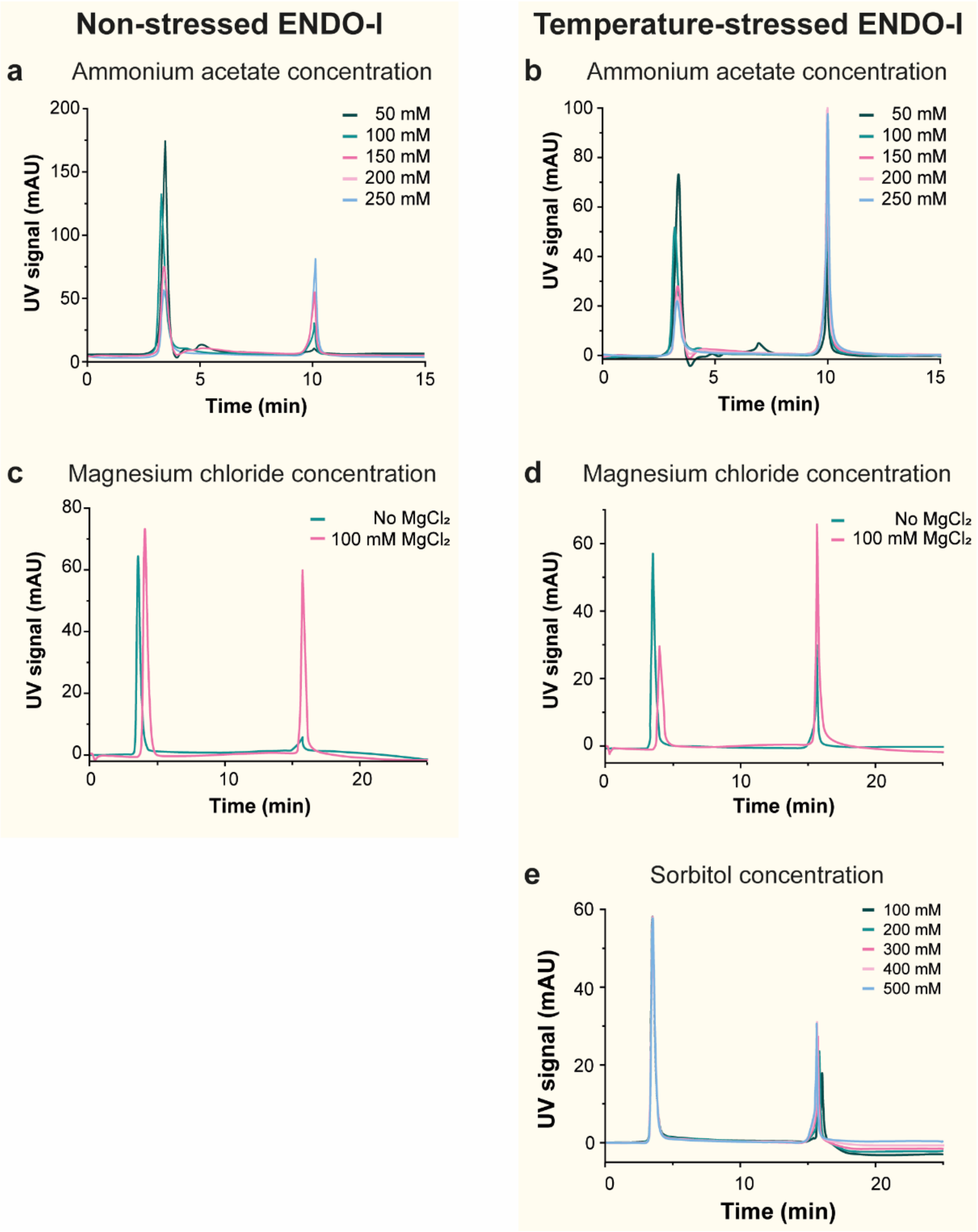

Figure S8. Development of the BAC method, including optimization of the buffer salt concentration, analysis of the effect of additional salts and evaluation of required sorbitol concentrations for elution. Overlay of UV-chromatograms measured with different concentrations of ammonium acetate in the mobile phase (i.e., 50, 100, 150, 200, and 250 mM) of non-stressed ENDO-I (**a**) and ENDO-I stored at 40°C for 20 weeks (**b**). Overlay of UV-chromatograms measured with 100 mM $MgCl_2$ or without $MgCl_2$ in the mobile phase of non-stressed ENDO-I (**c**) and ENDO-I stored at 40°C for 20 weeks. (**d**) Overlay of UV-chromatograms using different sorbitol concentrations (i.e., 100, 200, 300, 400, and 500 mM) to elute the thermal stressed ENDO-I (**e**). Besides optimization of these parameters, the gradient time was also shorted to minimize the exposure time of ENDO-I to the high pH of the mobile phase. In the optimized method (chromatogram a and b), the binding peak elutes at 10 min instead of 15 min for the non-optimal method (chromatogram c, d and e). The peak areas of the nonbinding and binding peaks of the measurements can be found in **Table S4**.

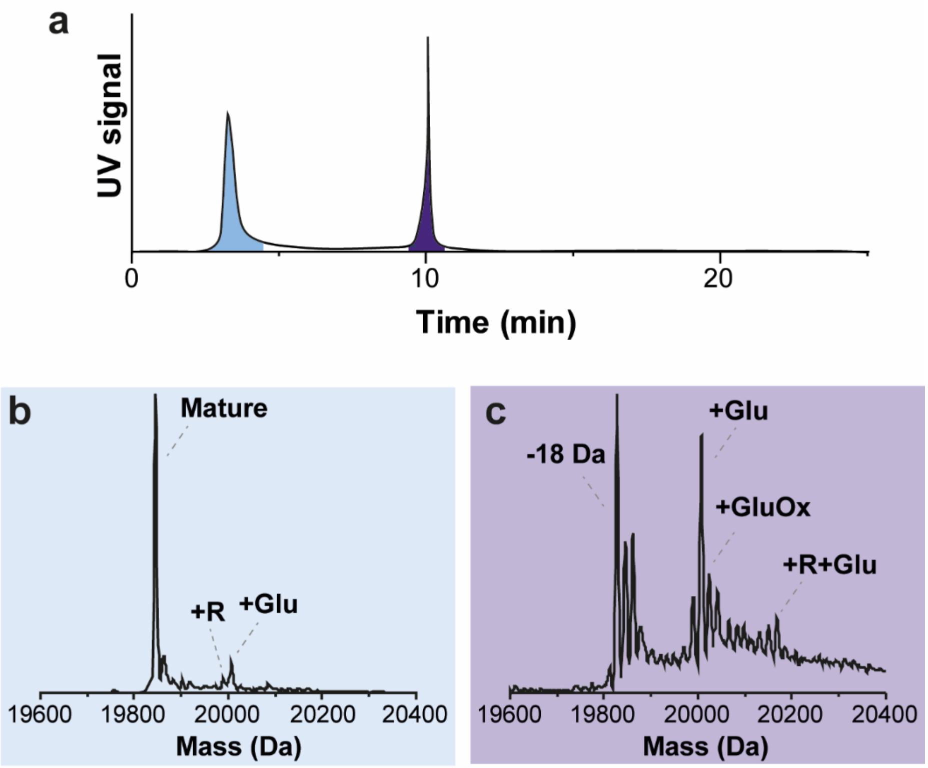

Figure S9. (**a**) BAC-UV chromatogram of temperature stressed ENDO-I (20 weeks at 40 °C). The indicated peaks were collected and analyzed with SEC-MS. (**b**) The mass spectrum of the nonbinding peak indicated with yellow in the UV chromatogram. Minor amount of glycated proteoforms are detected. (**c**) Mass spectrum of the binding peak indicated with green in the UV-chromatogram.

Table S1. Activity, protein concentration, specific activity and recovery of the ENDO-I samples after separation with SEC, IEX and BAC. The reported values for the activity and protein concentration are an average of three measurements. The specific activity was calculated by dividing the activity (U/mL) by the protein concentration (mg/mL). The recovery of SEC was calculated based on the total activity of the start material, while the recovery of IEX and BAC was calculated with the total activity of the SEC-separated ENDO-I peak.

Sample	LC mode	Peak	Activity (U/mL)	Protein conc. (mg/mL)	Specific activity (U/mg)	Volume (mL)	Total activity (U)	Recovery (%)
Non-stressed ENDO-I	-	ENDO-I start	4790	31	157	0.5	2395	-
	SEC	HMWS	-	-	-	-	-	-
		Gluco-amylase	2.29	2.7	0.84	1.0	2.29	0
		ENDO-I	1287	4.9	262	1.5	1930	81
		LMWS	11.7	0.0	∞	1.0	11.7	0
							**Total recovery (%)**	81
	IEX	+ R	23.7	0.1	274	1.0	23.7	6
		Mature	268	1.1	254	1.0	268	69
		+ GluOx	-	-	-	-	-	-
		+ 222Da	0.26	0.0	65.0	2.0	0.5	0
							**Total recovery (%)**	75
	BAC	Non-binding	47.3	0.2	305	2.0	94.7	74
		Binding	-	-	-	-	-	-
							**Total recovery (%)**	74
Temp. stressed ENDO-I (4 weeks)	-	ENDO-I start	4080	31	130	0.5	2040	-
	SEC	HMWS	8.08	0.0	-	1.0	8.08	0
		Gluco-amylase	14.5	2.5	5.79	1.0	14.5	1.0
		ENDO-I	974	4.2	230	1.5	1462	72
		LMWS	14.0	0.6	22.1	1.5	21.0	1.0
							**Total recovery (%)**	74
	IEX	Mature	200	0.9	236	0.9	180	62
		+ GluOx	23.0	0.1	227	1.1	25.3	9
		+ 222Da	0.21	0.0	42.0	2.0	0.42	0
							**Tot recovery (%)**	71
	BAC	Non-binding	32.3	0.1	231	2.0	64.6	66
		Binding	6.43	0.0	165	2.0	12.9	13
							**Tot recovery (%)**	79
Temp. stressed ENDO-I (20 weeks)	-	ENDO-I start	2997	32	94.3	0.5	1498	-
	SEC	HMWS	19.3	0.0	-	1.0	19.3	1
		Gluco-amylase	28.6	2.3	12.6	1.0	28.6	2
		ENDO-I	653	3.4	191	1.5	980	65
		LMWS	11.0	0.6	17.7	1.5	16.5	1
							**Tot recovery (%)**	69
	IEX	Mature	91.7	0.4	219	1.1	101	48
		+ GluOx	20.3	0.1	192	1.2	24.4	12
		+ 222Da	0.33	0.0	-	2.0	0.49	0

							**Tot recovery (%)**	60
	BAC	Non-binding	17.2	0.1	224	2.0	34.4	49
		Binding	5.89	0.0	130	2.0	11.8	17
							**Tot recovery (%)**	66

Table S2. The retention time (RT) and relative peak area (Rel. area) with standard deviation (SD) of identified proteoforms with SEC, IEX, and BAC. The reported values are an average of three measurements.

LC mode	Peak assignment	Non-stressed ENDO-I				Stressed ENDO-I (4 weeks)				Stressed ENDO-I (20 weeks)			
		RT (min)	SD	Rel. area (%)	SD	RT (min)	SD	Rel. area (%)	SD	RT (min)	SD	Rel. area (%)	SD
SEC	HMWS	-	-	-	-	8.4	0.0	2.3	0.1	7.7	0.0	11.6	0.1
	Glucoamylase	9.5	0.0	37.6	0.0	9.5	0.0	31.3	0.1	9.4	0.0	28.2	1.2
	ENDO-I	13.3	0.0	61.2	0.1	13.3	0.0	50.9	0.2	13.3	0.0	40.8	1.6
	LMWS	17.5	0.0	1.2	0.1	17.6	0.0	15.5	0.0	17.7	0.0	23.2	1.0
IEX	+R	3.7	0.0	2.0	0.1	3.8	0.0	3.1	0.2	3.9	0.0	3.4	0.2
	Mature (and + Glu)	4.9	0.0	89.8	0.1	5.0	0.0	73.9	0.3	5.0	0.0	57.9	0.8
	+ GluOx	6.6	0.0	1.1	0.1	6.6	0.0	10.9	0.2	6.6	0.0	22.3	0.2
	+ 222 Da	8.9	0.0	4.5	0.1	9.0	0.0	3.6	0.1	9.0	0.0	3.4	0.1
BAC	Non-binding	3.2	0.0	95.9	2.2	3.2	0.0	71.9	0.2	3.2	0.0	59.5	2.2
	Binding	10.9	0.0	4.1	2.2	10.9	0.0	28.1	0.2	11.0	0.0	40.5	2.2

Table S3. Analysis of specific activity differences between samples and statistical analysis of the measured specific activities of the non-separated materials and the fractions of SEC, BAC, and AEX. ENDO 0 corresponds to the non-stressed material, ENDO 4 is the 4-weeks temperature stressed material and ENDO 20 is the 20-week temperature stressed material. For the IEX fractions is reported which peaks are compared, where M is mature enzyme, R is mature enzyme with additional arginine, and GluOx is the glycoxidized variant. For the BAC fractions is indicated whether the non-binding (NB) or binding (B) peaks specific activity are compared. The reported differences are calculated from the average specific activities. All significant differences (using a 90% confidence interval) are marked in green. Details on the type of test can be found the methods section.

LC mode	Samples compared	Difference in specific activity	z-value (4 Degrees of Freedom)	p-value	Significant difference with 90% confidence level
Non-separated	ENDO 0 and ENDO 4	-26.3	-8.684	< 0.005	yes
	ENDO 0 and ENDO 20	-62.3	-20.16	< 0.005	yes
	ENDO 4 and ENDO 20	-36.0	-34.29	< 0.005	yes
SEC	ENDO 0 and ENDO 4	-37.0	-0.9321	0.404	no
	ENDO 0 and ENDO 20	-73.0	-2.348	0.079	yes
	ENDO 4 and ENDO 20	-36.0	-1.215	0.292	no
IEX	ENDO 0 (M) and ENDO 4 (M)	-17.0	-2.173	0.096	no
	ENDO 0 (M) and ENDO 20 (M)	-34.7	-7.109	0.002	yes
	ENDO 4 (M) and ENDO 20 (M)	-17.3	-2.391	0.075	yes
	ENDO 0 (M) and ENDO 0 (R)	20.7	-1.941	0.124	no
	ENDO 4 (M) and ENDO 4 (GluOx)	-10.3	-1.476	0.214	no
	ENDO 20 (M) and ENDO 20 (GluOx)	-26.0	-5.829	0.004	yes
BAC	ENDO 0 (NB) and ENDO 4 (NB)	-106	-1.752	0.163	no
	ENDO 0 (NB) and ENDO 20 (NB)	-89.3	-2.330	0.080	yes
	ENDO 4 (NB) and ENDO 20 (NB)	16.1	0.3417	0.775	no
	ENDO 4 (B) and ENDO 20 (B)	-38.0	-6.788	0.003	yes
	ENDO 4 (NB) and ENDO 4 (B)	-40.3	-0.8469	0.440	no
	ENDO 20 (NB) and ENDO 20 (B)	-94.0	30.41	0.000	yes

Table S4. Assignment of proteoforms separated with IEX-MS for the non-stressed and temperature-stressed (4 and 20 weeks) ENDO-I. Assigned proteoforms indicated with * are confirmed with MS/MS detection. Abbreviations: Glu = glycation, Ox = oxidation, PYRUS = Pyruvic acid N-terminal serine, R = additional N-terminal arginine, S = additional N-terminal serine, V= additional N-terminal valine.

Sample	RT (min)	Mass (Da)	Intensity	Theo. mass (Da)	Δ (Da)	Assignment	MS/MS
Non-stressed ENDO-I	5.2	19994.7	195298	19995.0	-0.4	+R	
		20081.7	24822	20082.1	-0.4	+RS	
		20181.6	25959	20181.3	0.3	+RSV	
	6.5	19838.4	4459105	19838.9	-0.5	Mature	*
		20000.4	621859	20001.0	-0.6	+Glu	*
	8.4	20217.1	26331	-	-	+R +222Da	
		20304.0	10371	-	-	+RS +222Da	
	10.7	20060.7	556180	-	-	+222Da	*
Temperature stressed ENDO-I (4 weeks)	4.4	19820.3	61868	19820.8	-0.5		
	5.1	19994.7	57382	19995.0	-0.4	+R	
		20082.4	9383	20082.1	0.3	+RS	
		20157.2	23927	20157.2	0.0	+R +Glu	
		20180.7	11624	20181.3	-0.5	+RSV	
	6.5	19838.3	1531246	19838.9	-0.5	Mature	*
		20000.3	564383	20001.0	-0.7	+Glu	*
	7.6	19911.2	21032			n.a.	
		20016.7	109120	20017.0	-0.3	+GluOx	*
	7.9	20042.1	7871	-	-	n.a.	
		20217.0	21557	-	-	+R+222Da	
		20303.5	11852	-	-	+RS+222Da	
	10.1	20060.6	312804	-	-	+222Da	
Temperature stressed ENDO-I (20 weeks)	4.3	19820.3	182462	19820.8	-0.5		
	4.9	19994.6	64449	19995.0	-0.5	+R	
		20157.7	14807	20157.2	0.5	+R +Glu	
		20181.3	17745	20181.3	0.0	+RSV	
	6.3	19838.2	1480553	19838.9	-0.7	Mature	*
		20000.0	198885	20001.0	-1.0	+Glu	*
	7.1	19910.8	55549	-	-	n.a.	
		20016.6	196547	20017.0	-0.4	+GluOx	*
	7.9	20042.5	21306	-	-	n.a.	
		20216.8	20105	-	-	+R+222Da	
		20304.1	11771	-	-	+RS+222Da	
	10.1	20060.4	142376	-	-	+222Da	*

Table S5. Mass difference of the glycated and glycoxidized variants of ENDO-I determined by IEX-CID-MS/MS analysis.

Modification	Fragment	*m/z* mature	*m/z* modification	Difference
+Glu	SAGINYVQ	833.4161	995.4204	162.0043
	SAGINYVQN	947.4522	1109.4814	162.0292
	SAGINYVQNY	1110.5183	1272.5433	162.0250
	SAGINYVQNYN	1224.5553	1386.5628	162.0075
			**Average**	162.0170 ± 0.0124
+GluOx	SAGINYV	705.3585	883.4542	178.0957
	SAGINYVQ	833.4085	1011.5159	178.1074
	SAGINYVQN	947.4491	1125.5591	178.1100
	SAGINYVQNY	1110.5098	1288.6295	178.1197
			**Average**	178.1080 ± 0.0099

Table S6. Retention times and peaks areas of the different measurements for the BAC method optimization for the non-stressed and temperature stressed ENDO-I. The corresponding UV chromatograms recorded at 280 nm can be found in **Figure S5**.

Mobile phase component	Concentration (mM)	Non-stressed ENDO-I				Stressed ENDO-I (weeks)			
		Non-binding peak		Binding peak		Non-binding peak		Binding peak	
		RT (min)	Rel. area (%)	RT (min)	Rel. area (%)	RT (min)	Rel. area (%)	RT (min)	Rel. area (%)
Ammonium acetate	50	3.4	100	-	-	3.4	77	10.0	23
	100	3.3	83	10.0	17	3.4	48	10.0	52
	150	3.3	64	10.0	36	3.3	35	10.0	65
	200	3.3	53	10.0	47	3.3	29	10.0	72
	250	3.3	49	10.0	51	3.3	26	10.0	74
Magnesium chloride	0	3.5	92.	15.7	8	3.5	74	15.7	26
	100	4.0	58	15.7	42	4.0	40	15.7	60
Sorbitol	100	-	-	-	-	3.5	78	16.1	22
	200	-	-	-	-	3.5	74	15.9	26
	300	-	-	-	-	3.5	74	15.8	26
	400	-	-	-	-	3.5	74	15.7	26
	500	-	-	-	-	3.5	74	15.7	26
